# Hepatitis E Virus (HEV): Identification of Subtypes 3b and 3m in Wild Boar Population in Liguria Region, Italy

**DOI:** 10.3390/pathogens11111236

**Published:** 2022-10-26

**Authors:** Roberta Battistini, Laura Serracca, Elisabetta Razzuoli, Valeria Listorti, Lisa Guardone, Monica Dellepiane, Nicola Pussini, Francesco Brunelli, Simone Peletto, Chiara Beltramo, Carlo Ercolini, Chiara Masotti

**Affiliations:** 1 Department of La Spezia, Istituto Zooprofilattico Sperimentale del Piemonte, Liguria e Valle d’Aosta, Via degli Stagnoni 96, 19100 La Spezia, Italy; 2 Department of Genoa, Istituto Zooprofilattico Sperimentale del Piemonte, Liguria e Valle d’Aosta, Piazza Borgo Pila 39/24, 16129 Genoa, Italy; 3 Department of Savona, Istituto Zooprofilattico Sperimentale del Piemonte, Liguria e Valle d’Aosta, Via Martiri 6, 17056 Savona, Italy; 4 Department of Imperia, Istituto Zooprofilattico Sperimentale del Piemonte, Liguria e Valle d’Aosta, Via Nizza 4, 18100 Imperia, Italy; 5 Department of Turin, Istituto Zooprofilattico Sperimentale del Piemonte, Liguria e Valle d’Aosta, Via Bologna 148, 10154 Turin, Italy

**Keywords:** hepatitis E virus, zoonoses, wild boar, HEV genotype 3, phylogeny

## Abstract

The wild boar is an important natural reservoir for the zoonotic transmission of the hepatitis E virus (HEV) around the world. In particular, HEV genotypes 3 and 4 are an emerging problem in industrialized countries, as the number of wild boars has increased, and their territory is ever closer to farms and populated areas. This study describes the HEV prevalence and geographic circulation among wild boar populations in the Ligurian region (Italy) during the period 2019–2022. Liver samples from 849 wild boars were analyzed for HEV RNA using real-time RT-PCR; positive samples were then subjected to sequencing and phylogenetic analysis. Overall, 6.7% of the wild boars were positive for HEV RNA; however, in the last two years, the percentage of positive animals almost doubled. Phylogenetic analysis showed that wild boar HEV sequences belonged to genotype 3 and clustered within subtypes 3a and 3c, and, for the first time in Italy, subtypes 3b and 3m were identified. Interestingly, 13 sequences could not be assigned to a recognized subtype. Furthermore, the results showed different circulations of identified subtypes across the territory. These findings increase the knowledge of HEV-3 heterogeneity in Italy and describe the role of wild boars in maintaining an active viral circulation in the environment.

## 1. Introduction

Hepatitis E infection is recognized worldwide as an emerging public health issue [1]. Hepatitis E virus (HEV), the causative agent of human hepatitis E, is a small (27–34 nm) single-stranded, positive-sense, quasi-enveloped RNA virus, classified in the genus *Paslahepevirus* belonging to the *Hepeviridae* family, subfamily *Orthohepevirinae* [2]. Its genome is approximately 7.2 kb and contains three overlapping open reading frames (ORF1, ORF2, and ORF3). ORF1 encodes nonstructural proteins, including the viral replicase proteins. ORF2 encodes a 660-amino acid viral capsid, which is responsible for the induction of immune responses. ORF3 encodes a small multifunctional protein involved in releasing new virions [3]. The *Paslahepevirus* genus is divided into two species (*Paslahepevirus balayani* and *Paslahepevirus alci*); only *Paslahepevirus balayani* infect humans. The *Paslahepevirus balayani* species includes eight different genotypes (HEV 1−8): genotypes 1 and 2 primarily infect only humans and are the cause of most of the epidemic events in Asia, Africa, and Mexico. Genotypes 3 and 4 infect humans and animals, including pigs and wild boars [2]. Genotype 3 circulates in the United States, Japan, and several European countries, where it is the most frequently detected genotype, while genotype 4 is typical in Asia [4]. HEV-3 and HEV-4 can be transmitted from animal to animal via the fecal–oral route or from animal to humans through the consumption of contaminated foods of animal origin. HEV is responsible for a self-limiting form of acute infectious hepatitis, which usually resolves within 2–6 weeks. [5,6]. In Europe, between 2005 and 2015, the number of notified cases of hepatitis E showed a progressive 10-fold increase, with more than 21,000 confirmed cases overall and a total of 28 deaths, underlining the relevance and emerging nature of this zoonotic infection [7]. Most of the cases arise from foodborne infections caused by the zoonotic HEV genotypes HEV-3 and HEV-4 [8]. Several human cases have been linked to the consumption of raw or undercooked animal products of both pork (liver sausages) and wild boar meat [5,6,9]. Additionally, in Italy, since 2007, an increasing number of human cases have been reported. The annual number increased from 12 in 2007 to 49 in 2018; the increasing trend continued in 2019, when an epidemic peak of 102 cases was observed, linked to several outbreaks that have occurred in the central regions (Marche, Lazio, and Abruzzo) and linked to the consumption of pork and wild boar [10,11,12]. Recently, however, increasing cases of indigenous origin have been described as being associated with direct or indirect contact with pigs, wild boars, and other animals, which represent the main asymptomatic reservoir of the virus [7]. Among wild species, the wild boar is considered the main reservoir of HEV and plays an important role in the transmission of the virus in Europe [13]; in Italy, the prevalence of HEV in wild boars ranges between 2.45% and up to 52.5% depending on the region studied [14,15,16,17,18,19,20,21]. It has been experimentally shown that HEV-infected wild boars can transmit the infection to other animals, such as pigs [22,23]. This has a significant impact, especially in countries where extensive pig farming is widespread, because contact between domestic pigs and sympatric species is facilitated, thus increasing the risk of interspecies transmission [24,25,26]. Wild boar hunting and the consumption of wild boar meat are increasing, leading to greater chances of direct exposure to wild boar zoonoses [27]. Hunters, slaughterhouse workers, and veterinarians are primarily at risk [28]. Direct transmission during hunting, especially during evisceration and skinning, may lead to infection through direct contact with the organs and tissues of infected wild boars [29,30,31]. Knowledge of the health status of wildlife is therefore essential, especially as wild animal populations are increasing across Europe [32]. Furthermore, circulation of HEV between wild species other than wild boar has been reported in several studies as an additional reservoir for the virus. In Italy, HEV-3 has been found in red deer (from 0.8% to 5.6%) [33,34], chamois (1.2%) [33], wolves [35], rats [36] sheep (21.6%) [37], goats (11.4%) [37], roe deer (3.1%) [37], alpine ibex (6.3%) [37], and wild rabbits (38.5%) [34]. Epidemiological surveillance aims to provide a comprehensive view of the health status of animal populations, as well as ensuring effective risk assessment. In Italy, the regional authorities can issue local regulations on the management and protection of all wild species and can implement control plans to ensure the general health of the wildlife and the monitoring of their territory. 

In this study, we investigated the prevalence of HEV in wild boars hunted in the Liguria region (northwest Italy) in the period of 2019–2022. To determine virus circulation and characterize the detected strains, liver samples were tested for HEV using real-time reverse-transcription PCR (RT-qPCR) and were genotyped with sequencing and phylogenetic analysis. 

## 2. Results

Overall, HEV RNA was detected in 57 out of 849 (6.7%, CI 95% 5.2–8.6) tested wild boar liver samples during three hunting seasons (2019–2022). From 2019 to 2022, a virus prevalence of 4.2%, 8.2%, and 7.7%, respectively, was detected (Table 1). 

**Figure 1:**
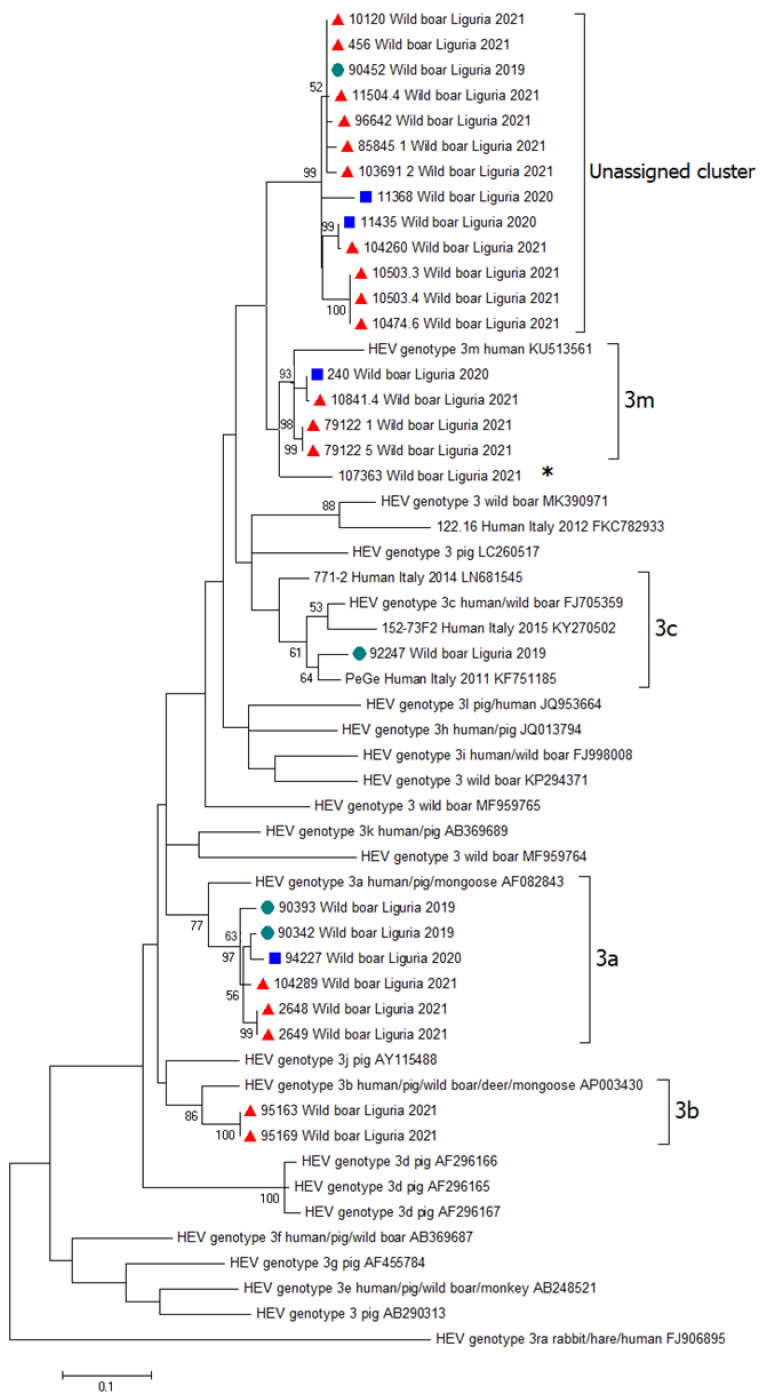
Phylogeny inferred by maximum likelihood (ML) analysis obtained by an alignment of 348 nucleotides, covering the 5′ end of ORF2 gene of HEV. Samples identified in the study are indicated based on the years of collection, as follows: ● 2019; 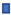 2020; ▲2021. The phylogenetic tree includes the reference sequences proposed by Smith et al. [39] 0 for HEV-3 subtypes, and sequences from Italian human patients available in GenBank. * indicates the sequence divergent from subtype 3m. Bootstraps (1000 replicates) values >50 are shown at the internal nodes. The length of each pair of branches represents the distance between sequence pairs. The scale bar represents the percentage of nucleotide differences.

**Figure 2:**
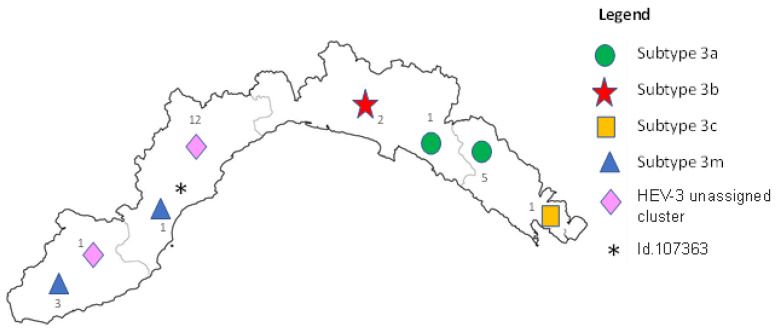
Geographic distribution and the number of detected HEV subtypes in the Ligurian region.

Positive real-time PCR samples were analyzed using RT-nested PCR for sequencing and genotyping. Sequences of appropriate quality for phylogenetic analysis could be obtained for 27 out of 57 HEV samples; the other positive samples could not be genotyped for lack of amplification, likely due to the low viral titer (high Ct at the diagnostic real-time assay) or because of low-quality sequences, even after several attempts. The phylogenetic tree topology showed that all wild boar HEV sequences belonged to genotype 3; specifically, six sequences clustered within subtype 3a, two within subtype 3b, one within subtype 3c, and four within subtype 3m. All sequences assigned to a specific subtype were checked for nucleotide p-distances (Table S1), which were below the cut-off (0.093 nt substitutions/site) proposed by Nicot et al. [38] for subtype definition. One sequence (id. 107363) showed divergence from subtype 3m and was not assignable due to ambiguous positioning and nucleotide p-distances of 0.074 nt substitutions/site for both 3m and 3c subtypes. Interestingly, thirteen isolates formed a putative novel cluster not assignable to any known subtype, even though the p-distance fell exactly on the cut-off (0.093) when calculated for subtype 3m. A detailed phylogenetic tree is reported in Figure 1. Sequence homologies, determined for strains within each detected HEV subtype, were: 91.8–100% (3a), 98.7% (3b), 96.7–100% (3m), and 92.8–100% (unassigned cluster). The geographic distribution of HEV-3 subtypes among wild boars in the Liguria region showed a different circulation across the territory: in the western area, HEV strains belonging to subtype 3m and to the unassigned cluster were prevalent, while in the eastern part, HEV subtype 3a was present as well as subtype 3c (Figure 2). Finally, in this study, HEV subtypes 3b and 3m were identified for the first time in the country. 

## 3. Discussion

In this study, 849 samples collected from wild boars in the Liguria region were analyzed for the presence of HEV RNA using real-time RT-PCR, resulting in an overall prevalence of 6.7%. In the analyzed period (2019–2022), the percentage of positive animals showed an increasing trend in the last two years: up to 8.2% and 7.7%. In 2019–2020, HEV prevalence in the wild boar population was lower (4.2%) and similar to the findings of the previous years for the same area: 4.3% (12/280) in 2018–2019 and 4.6% (13/280) in 2017–2018 [40]. The HEV RNA prevalence observed in our study in the last two years is similar to the prevalence reported in Europe (8.7%; 95% CI, 6.7–11) and, in particular, to that reported in southern Europe (8%; 95% CI, 5–12) and western Europe (6%; 95% CI, 4–10) [41]. Our data, in contrast, are among the lowest compared with results obtained in the previous studies conducted on animals hunted in Italy, with an HEV positivity ranging from 2.45% (Umbria and Marche regions) to 52.2% (Lazio region) [14,15,16,17,18,19,20,21]. In the last study years, an increase in HEV detection rates in wild boars was also reported in France, Germany, Italy, the Czech Republic, Lithuania, Portugal, Poland, and Spain [42]. EFSA BIOHAZ [9] reported a 10-fold increase in reported HEV human cases (HEV-3) among western European countries. Foodborne transmission of HEV appears to be a major route in Europe; pigs and wild boars are the main sources of HEV. In this study, phylogenetic analyses showed the presence of subtypes HEV-3a, 3b, 3c, and 3m. The subtypes HEV-3a and 3c were already present in this area, as reported in previous studies. In particular, HEV-3c has been present in the area since 2012 [43] and HEV-3a since 2017 [40]. HEV-3b, on the other hand, was found, for the first time, in the last hunting season analyzed (2021–2022), while the 3m subtype has appeared from the 2019–2020 hunting season onward. The HEV-3b subtype was reported for the first time in wild boars in Germany in 2015 [44], and then in two studies on pigs in Slovenia and Serbia in 2017 and 2019, respectively [45,46]. The HEV-3m subtype was detected in a Spanish patient for the first time in 2011 [47] and later in humans in other European countries [38]; it was detected in wild boars in Spain in 2015 [48]. In a human case in Sweden, transmission through contaminated meat, water or direct contact with wild boar was hypothesized [49]. In Italy, HEV-3b and 3m have not been found in any wild boar sample so far. Therefore, to the best of our knowledge, this is the first report in the country. In the previous study [43], in addition to subtype 3c, subtypes 3e and 3f were identified. The HEV-3f subtype was also found in the subsequent study relating to the years 2017–2019 [40], but not in this current study; the HEV-3e subtype was no longer found. In Europe, HEV-3c has recently emerged as predominant in humans, pigs, and wild boars [13,49]. A close phylogenetic relationship may be observed between the only 3c sequence detected in this study, from a wild boar in 2019, and a human isolate from a male patient with acute hepatitis E dating back to 2011. Despite the time distance, interestingly, this patient was hospitalized in Genoa (Liguria region) and figatelli. French food was identified as the probable source of infection at that time (unpublished). In Italy, HEV-3f has been reported as the most common subtype detected in wild boar, followed by HEV-3c; HEV-3e; other rare subtypes reported less frequently, such as HEV-3a; and others that remain unclassified [50]. HEV-3a has been reported in Europe (Germany, Austria, Croatia, Hungary, Belgium, and Poland) [51,52,53,54], although it can still be considered rare. In Italy, the HEV-3a has been recently reported in wild boar [17,55]. 

In our study, four of the isolates formed a cluster with the sequence KU513561; this isolate was identified in Spain in 2011 from a human sample and was previously classified as an unassigned subtype named HEV 3chi-new. Recently, this new subtype was named 3m, mainly containing sequences from European strains [39]. One sequence (id. 107363) diverged from subtype 3m, forming a separate branch; the results of p-distance analysis showed the same values of nucleotide substitutions per site when comparing this strain with subtypes 3m and 3c, thus confirming the ambiguous nature of this isolate. Moreover, 13 isolates grouped into a separate cluster that were notably phylogenetically distinct from known subtypes, as defined by the recently proposed reference sequences used for subtyping. P-distance analysis did not help, because the obtained 0.093 value for subtype 3m fell exactly on the suggested cut-off for subtype definition [38]. Therefore, whether these strains represent a novel HEV subtype remains to be elucidated, and further molecular investigations are needed, possibly on the whole viral genome, to increase the phylogenetic signal. The results from the phylogenetic analysis suggest that the virus is constantly circulating and that it is increasing over time (years) within the same geographic area. Furthermore, differences seem to exist in the studied geographic area of HEV subtype circulation. Similar studies identified different subtypes based on the sampling location [56,57]. In our study, the observed differences in the geographic distribution of HEV subtypes could have been influenced by the presence of a large motorway on the territory that crosses the central part of the region, dividing it into two parts. This road represents a physical barrier to the movement of wild boars, which may reduce the mixing of eastern and western populations and vice versa. According to our data, HEV-3a is circulating in the eastern part of the Ligurian region; the same subtype was found in wild boars in the Parma province located about 80 km away from our findings in the neighboring region (Emilia Romagna) [58]. Moreover, the HEV-3c subtype was found in a wolf from the same geographic area, where it was found through our surveillance of wild boars (La Spezia, Liguria region) [36], highlighting both the circulation in this area and in other wild species. In summary, it can be highlighted that the subtypes 3a and 3c have persisted for a longer time in the investigated area, unlike the other subtypes found. This finding is in line with a recent large study including wild boars from Germany that indicated that dominant HEV subtypes circulate in a specific area over a long period of time, whereas minor subtypes were only detectable for a short time [57]. The geographic peculiarities of the Ligurian region, covered by mountains on over 65% of its territory and by hills for most of the remaining surface, together with the short distance between coastal and mountainous areas, contribute to contact between humans and domestic and wild animals. For these particularities, constant and long-term surveillance and control measures should be in place, in particular for a multihost zoonosis, and control of the emergence of new subtypes, which represent a new zoonotic risk factor. Although there are no intensive pig farms in the investigated area and therefore transmission between swine and wild boars is very limited, the neighboring regions (Emilia Romagna, Piedmont, and Lombardy) host intensive pig farms. Wild boar has been shown to have an average dispersed capacity of about 17 km, but this can occasionally increase to 105 km [59] and 250 km [60]. Greater efforts should be made when establishing preventive and biosecurity measures or when developing surveillance programs to minimize the circulation of diseases and, therefore, transmission. Constant monitoring of the health of the circulation of wildlife is essential. 

## 4. Materials and Methods

### 4.1. Sampling 

**Figure 3:**
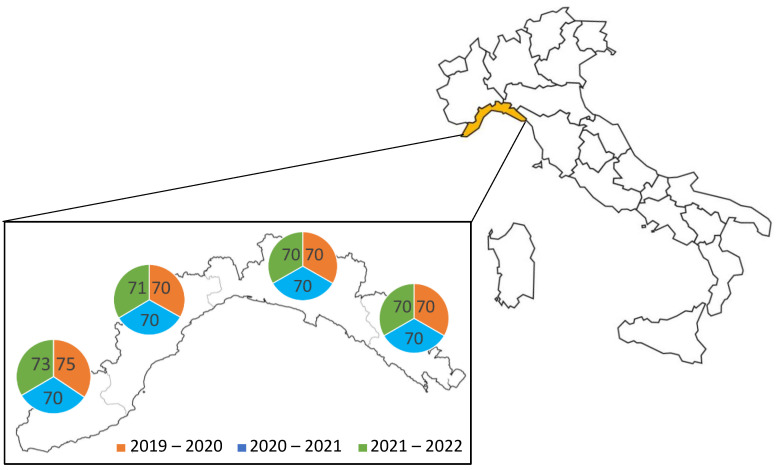
Map of localization of Ligurian region in Italy where the wild boars were hunted and distribution of collected samples during the period analyzed.

A total of 849 liver samples were collected from wild boars (*Sus scrofa scrofa*) during three hunting seasons (from October to January): in 2019–2020 (285 samples), 2020–2021 (280 samples), and 2021–2022 (284 samples) from both the Apennines and Alps areas of the Liguria region, in the northwest part of Italy (Figure 3). After collection, the samples were immediately analyzed or stored at −80 °C until analysis. 

### 4.2. Viral Recovery and RNA Extraction 

Tissue samples were manually excised and finely chopped with sterile surgical blades. Next, 25 mg aliquots of chopped tissue were added to 300 µL of sterile phosphate-buffered saline (pH 7.2) and then homogenized by a TissueLyser (QIAGEN, Hilden, Germany) for 2 min. Finally, samples were clarified by centrifugation at 14,000× *g* for 2 min. Nucleic acid was extracted from 200 µL of the supernatant using an IndiSpin Pathogen Kit (Indical, Bioscience), according to the manufacturer’s instructions. RNA was eluted in 100 µL and stored below −80 °C prior to real-time PCR analysis. Positive and negative controls were extracted in parallel with the liver samples. 

### 4.3. TaqMan Real-Time RT-PCR 

HEV real-time RT-PCR was performed using an RNA Ultrasense One-Step qRT-PCR system (Invitrogen, Carlsbad, CA, USA) and primers and probe as previously described [61]. For each reaction, 10 µL of RNA was added to a mix containing 1× PCR buffer RNA Ultrasense reaction mix, 0.25 µM primers, 0.1 µM probe, 1 µL RNA Ultrasense enzyme mix, and water to a total volume of 20 µL. The amplification conditions were as follows: reverse transcription for 15 min at 50 °C, inactivation at 95 °C for 2 min and 45 cycles at 95 °C for 10 s, 55 °C for 20 s, and 72 °C for 15 s [62]. Negative and positive controls were included in all amplifications. Reactions were carried out using a Biorad CFX96TM Real-Time PCR thermocycler (Biorad, Hercules, CA, USA). 

### 4.4. Sequencing and Phylogenetic Analysis 

The phylogenetic analysis was performed by comparing the 5′ region of the ORF2 gene, targeting a variable region that provides a phylogenetic signal comparable to full genome analysis [63]. RT was performed at 42 °C for 60 min with Superscript II reverse transcriptase (GIBCO-BRL) using reverse primer 3157N. A nested PCR assay was performed using an external primer set (3156N forward, 3157 reverse) for a first amplification round (710 bp) and an internal primer set (3158N forward, 3159 reverse) for the second round (348 bp) [64]. The assay was carried out in a final volume of 50 μL with 1× Maxima Hot Start Taq buffer, 1.5 mM MgCl_2_, 0.2 mM of each dNTP, 0.2 μM of each primer, and 1U of Maxima Hot Start Taq DNA Polymerase (Thermo Fisher Scientific, Waltham, MA, USA). The following thermal conditions were applied: activation of Taq polymerase at 95 °C for 4 min, followed by 40 cycles of denaturation at 95 °C for 1 min; annealing at 60 °C for 1 min; extension at 72 °C for 2 min; and final elongation at 72 °C for 7 min. Amplification products were checked using electrophoresis on 2% agarose gel, purified with Extract me DNA Clean-up (Blirt, Gdańsk, Poland) and sequenced using a BrilliantDye Terminator v3.1 cycle sequencing kit (NimaGen, Nimega, The Netherlands). The amplicons were purified with a DyeEx 2.0 Spin Kit (Qiagen, Hilden, Germany) and run on a 3130xl Genetic Analyzer (Life Technologies, Carlsbad, CA, USA).

The final dataset, including the study samples and 26 GenBank reference sequences, was aligned, and a phylogenetic tree was constructed using MEGA7 software with the maximum likelihood method. For phylogenetic inference, the best nucleotide substitution model was estimated using jModelTest2 [65] and Tamura-Nei, using a discrete gamma distribution (G) and invariable sites (I). The statistical robustness and reliability of the branching order were confirmed with bootstrap analysis using 1000 reiterations. Nucleotide p-distances were calculated within MEGA7 by grouping sequences according to the tree topology; the number of base substitutions per site was estimated by averaging all sequence pairs between groups and reference sequences. 

The HEV sequences were submitted to NCBI GenBank under accession numbers OP687896–OP687922.

### 4.5. Statistical Analysis 

The prevalence of HEV by year was calculated with the 95% confidence interval (CI) (http://epitools.ausvet.com.au/content.php?page=home, accessed on 21 July 2022). 

## 5. Conclusions

Our study found an increasing trend in HEV prevalence in wild boar populations of the Ligurian region in northwest Italy in 2021 and 2022, and provides the first analysis of the genetic diversity and circulation dynamics of HEV in wild boars in this area. Moreover, the present survey is, to the best of our knowledge, the first showing the presence of the HEV subtype 3b in wild boars in Italy. These findings increase our knowledge of the HEV-3 heterogeneity in Italy and show the role of wild boars in maintaining an active viral circulation in the environment and as a possible source of HEV infection in other animals and humans. 

## Figures and Tables

**Table 1:** Prevalence of HEV RNA detected in liver samples by year and HEV subtype identified.

Hunting Season Year	No. Positive/ No. Tested	Prevalence (%) [95% CI]	Sequenced/ Positive	HEV Subtype (No. Identified)
2019–2020	12/285	4.2 [2.4–7.2]	7/12	3a(2), 3c(1), 3m(1), 3 UN ^1^ (3)
2020–2021	23/280	8.2 [5.5–12.0]	9/23	3a(3), 3m(1), 3 UN ^1^ (5)
2021–2022	22/284	7.7 [2.2–11.4]	11/22	3a(1), 3b(2), 3m(2),3 UN ^1^ (5), (1) ^2^
Total (2019–2022)	57/849	6.7 [5.2–8.6]	27/57	3a(6), 3b(2), 3c(1), 3m (4), 3 UN ^1^ (13), (1) ^2^

^1^ UN = unassigned cluster; ^2^: id. 107363.

## Data Availability

The HEV sequences have been submitted to NCBI GenBank. The accession numbers corresponding the sequences data are OP687896–OP687922.

## Supplementary Materials

The following supporting information can be downloaded at: https://www.mdpi.com/article/10.3390/pathogens11111236/s1, Table S1: Estimates of p-distance over sequence pairs between groups. The number of base substitutions per site from an average of all sequence pairs between groups are shown. Analyses were conducted using the maximum composite likelihood model. The analysis involved 43 nucleotide sequences. Codon positions included were 1st + 2nd + 3rd + noncoding. All positions containing gaps and missing data were eliminated. Evolutionary analyses were conducted in MEGA7 [66,67].
